# *Toxoplasma gondii* virulence in mice is determined by the pseudokinase ROP5B and countered by an IRG-GBP protein interplay

**DOI:** 10.3389/fimmu.2025.1593785

**Published:** 2025-07-09

**Authors:** Shishir Singh, Mateo Murillo-León, Aura María Bastidas Quintero, Florence Melbert, Klaus Pfeffer, Daniel Degrandi, Tobias Steinfeldt

**Affiliations:** ^1^ Institute of Virology, Medical Center University of Freiburg, Freiburg, Germany; ^2^ Faculty of Biology, University of Freiburg, Freiburg, Germany; ^3^ Faculty of Medicine, University of Freiburg, Freiburg, Germany; ^4^ Institute of Medical Microbiology and Hygiene, Medical Center University of Freiburg, Freiburg, Germany; ^5^ Center for Integrative Biological Signalling Studies (CIBSS), University of Freiburg, Freiburg, Germany; ^6^ Institute of Medical Microbiology and Hospital Hygiene, Heinrich-Heine-University Düsseldorf, Düsseldorf, Germany

**Keywords:** host cell resistance, co-evolution, IRG/GBP proteins, *T. gondii* virulence, pseudokinases

## Abstract

*Toxoplasma gondii* (*T. gondii*) virulence in mice depends on different multiprotein complexes that assemble at the parasitophorous vacuole membrane (PVM) of the parasite. Individual rhoptry proteins within these complexes inhibit different Immunity-Related GTPases (IRG proteins). The rhoptry pseudokinase ROP5 is a central element to achieve IRG-specific rhoptry kinase activity and/or efficient complex formation. The *rop5* locus of each of the canonical *T. gondii* strains encodes three major isoforms, ROP5A, ROP5B and ROP5C, and was shown to have the largest impact on virulence. By reverse genetics, we have generated *T. gondii* strains expressing either ROP5A, ROP5B or ROP5C in a RHΔ*rop5* genetic background and demonstrate that ROP5B is mainly responsible for heightened virulence of type I *T. gondii* in laboratory strains of mice. *In vivo* virulence correlates with diminished vacuolar IRG protein loading and parasite control *in vitro* only in presence of ROP5B but not ROP5A or ROP5C. Our results suggest that ROP5A and ROP5C isoforms might have co-evolved with IRG proteins or other host cell resistance factors in evolutionarily important intermediate hosts beyond *Mus musculus*. The same parasite effectors that inhibit IRG protein accumulation and function reduce the vacuolar amount of Guanylate Binding Proteins (GBP proteins). However, a parasite effector targeting a GBP protein at the PVM has not been described yet. Using two different approaches, Yeast Two-Hybrid analysis and Protein-fragment complementation assay, we here identified three heterologous IRG:GBP pairs, GBP6:Irgb10, GBP5:Irgb10, GBP5:Irgb6, and demonstrate that the accumulation of these GTPases at the PVM is interdependent. Our results offer a novel perspective on the IRG and GBP protein-mediated control of *T. gondii* infections and may further advance the investigation of GBP-specific *T. gondii* effectors.

## Introduction

*Toxoplasma gondii* (*T. gondii*) is a zoonotic parasite that can infect virtually all warm-blooded animals. Sexual recombination, however, occurs only in its primary host, represented by all felids. After excretion and sporulation, oocysts serve as source of infection for a variety of intermediate hosts. In mice, acute infection is readily controlled by innate and adaptive immune responses leading to encystment and establishment of a chronic infection. In North America and Europe, three main clonal lineages predominate (1, 2). Type I strains are usually lethal to conventional laboratory mice at low inocula (3), whereas type II and III strains are significantly less virulent (2). The population genetic structure of *T. gondii* in other parts of the world, particularly in South America (SA), is more diverse. Strains appear to undergo more frequent recombination and are referred to as atypical *T. gondii* strains. Most of these atypical strains are associated with high mortality rates in conventional laboratory mice and manifestation of human ocular toxoplasmosis (4–6).

Resistance against infection by *T. gondii* in mice is largely dependent on two families of IFN-γ-inducible proteins, the Immunity-Related GTPases (IRG proteins) and Guanylate Binding Proteins (GBP proteins). IRG and GBP proteins accumulate at the parasitophorous vacuolar membrane (PVM) of the parasite, a necessary prerequisite for vacuolar disruption and host cell death (7, 8). Compared with type II and III strains, IRG/GBP-mediated parasite control is significantly impaired in type I strain infections. *T. gondii* type I strains have evolved polymorphic effector proteins that are secreted from apical organelles into the host cell cytosol. They assemble into multiprotein complexes at the PVM to inactivate different IRG proteins. Inactivation of IRG/GBP proteins directly correlates with increased type I strain virulence. So far, four rhoptry proteins (ROPs), ROP5/ROP17/ROP18/ROP39, and two dense granules proteins (GRAs), GRA7/GRA60, have been identified to inactivate three IRG proteins (9).

Residues that are considered to be essential for enzymatic kinase activity (the catalytic triad) (10) are altered in several ROP2 superfamily members (11) and yet the largest impact on *T. gondii* virulence can be attributed to the pseudokinase ROP5. The *rop5* locus comprises a cluster of closely related polymorphic genes. While type I and type III strains share similar *rop5* alleles, type II strains carry a distinct set of alleles that is associated with significantly less virulence (12, 13). Three major isoforms, ROP5A, ROP5B and ROP5C, are encoded within the locus of each of the three canonical strains, each locus containing multiple copies (12). We could demonstrate that isoform ROP5B is the most important determinant of *T. gondii* type I virulence in cells derived from laboratory strains of mice (14). Different combinations of ROP5 isoforms A, B and C were tested for their significance in *T. gondii* virulence *in vivo* (12, 15), however, the contribution of ROP5B alone in this regard has not been addressed yet. Here, we investigated the dependence of *T. gondii* virulence on individual ROP5 isoforms in C57BL/6 (BL/6) animals.

Reciprocal host-parasite-interactions drive evolution and foster biological innovation. A higher parasite burden enhances the success for transmission but is often associated with increased virulence, leading to reduction in host fitness and favouring selection of enhanced defence mechanisms. This evolutionary arms race is exemplified in the case of type I *T. gondii* strain infections in wild-derived mice like CIM (*Mus musculus castaneus* from India), where *T. gondii* encounters an IRG system that is particularly polymorphic in the *IRGB* alleles (16). Irgb2-b1, the most polymorphic family member, sequesters type I ROP5B, thereby impairing its function and preventing the assembly of the IRG-related multiprotein complexes (14). However, CIM mice do not control infections with atypical *T. gondii* strains, which are genetically more diverse than the clonal lineages, and the genetic basis of enhanced virulence appears to be conserved, as ROP5 and ROP18 have been identified as important virulence components of different isolates from SA and Asia (17). In this study, we demonstrate using *T. gondii* type 10 VAND (18, 19) as an example that virulence of atypical *T. gondii* strains in CIM mice is based on the C-terminal polymorphic surface of genetically diverse ROP5 variants that allows escape from Irgb2-b1 recognition.

We have shown recently that binding of type I ROP5A and ROP5B to GBP5 provides *T. gondii* with a mechanism to inhibit inflammasome activation, possibly in cases where PVM protection is insufficient (20). GBP5 is the first GBP protein identified as target of a *T. gondii* effector. It remains unclear, however, whether a parasite effector can directly inhibit GBP accumulation at the PVM, or if this accumulation is reduced indirectly through the inhibition of IRG proteins by IRG-specific effectors. The latter would most likely involve an interaction between both GTPase families. To address a possible interplay between IRG and GBP proteins in PVM loading, we investigated the formation of heterologous interactions. We identified three IRG: GBP pairs, GBP6:Irgb10, GBP5:Irgb6 and GBP5:Irgb10, and show that accumulation at the PVM is interdependent. These data demonstrate that IRG and GBP proteins are not separate entities in the control of murine *T. gondii* infections. Additional IRG: GBP pairs may be identified in the future, providing further insights into the mechanisms underlying relocalisation of these GTPases to the PVM and thus advancing our understanding of *T. gondii* control.

## Materials and methods

### Cell culture

Mouse embryonic fibroblasts (MEF) and tail fibroblasts derived from C57BL/6 (BL/6) mice and human foreskin fibroblasts (HS27, ATCC; CRL-1634) were maintained by serial passage in DMEM, high glucose (Invitrogen Life Technologies) supplemented with 2 mM L-glutamine, 1 mM sodium pyruvate, 1x MEM non-essential amino acids, 100 U/ml penicillin, 100 mg/ml streptomycin (PAA) and 10% fetal calf serum (FCS, Anprotec).

For generation of *GBP5* ko bone marrow-derived macrophages (BMDMs), bone marrow cells were flushed from tibias and femurs using a 26 G needle. After filtration through a 100 µm cell strainer, cells were pelleted for 5 min at 450 g, resuspended in 1 mL of RBC lysis buffer (Qiagen) and incubated for 5 min at RT to remove erythrocytes. After addition of 9 mL of flush-medium, cell pellets (450 g, 5 min, RT) were resuspended in 10 mL RPMI medium supplemented with 40 ng/ml M-CSF (PeproTech) at 5 x 10^5^ cells/ml. Fresh containing M-CSF was added every two days for 6 days.

All cells were regularly tested by PCR for mycoplasma (21).

### Propagation of *T. gondii*

Tachyzoites of *T. gondii* GT-1, RHΔ*hxgprt*, RHΔ*rop5*, RHΔ*rop5*+*rop5*A, RHΔ*rop5*+*rop5*B, RHΔ*rop5*+*rop5*C and ME49 (clone B7-21) were cultivated in confluent monolayers of HS27 cells in DMEM medium containing high glucose supplemented with 100 U/ml penicillin, 100 mg/ml streptomycin and 2% FCS, harvested and immediately used for *in vitro* or *in vivo* infections or lysed for subsequent Western blot analysis.

### Postnuclear lysate preparation from extracellular tachyzoites

10 x 10^6^ extracellular *T. gondii* tachyzoites were lysed in NP-40-lysis buffer (0.1% NP-40, 150 mM NaCl, 20 mM Tris/HCl (pH 7.6), 5 mM MgCl_2_ supplemented with protease inhibitors) for 1 h under constant rotation at 4°C. Postnuclear lysates were subjected to Western blot analysis.

### Animal strains and infection conditions

BL/6 mice were obtained from certified breeders. Female and male mice with ages ranging from 6 to 8 weeks were infected intraperitoneally (i.p.) with 200 µl of sterile PBS containing freshly harvested tachyzoites of indicated *T. gondii* strains. Infected animals were monitored daily for 30 days. Relative weight loss was calculated based on the weight at the day of infection. Survivors were sacrificed at the indicated days post-infection and tested for sero-conversion by ELISA (22). Mice were kept under specific-pathogen-free conditions in the local animal facility (Department for Microbiology, Virology and Hygiene, Freiburg).

### Preparation of *T. gondii* DNA

Genomic DNA (gDNA) was prepared from *T. gondii* tachyzoites using the Quick DNA miniprep kit.

### Generation of transgenic *T. gondii* strains

For generation of RHΔ*rop5*+*rop5*A, RHΔ*rop5*+*rop5*B and RHΔ*rop5*+*rop5*C, 1 x10^7^ freshly egressed RHΔ*rop5* tachyzoites were pelleted for 15 min at 1,000 g, washed and resuspended in cytomix buffer (120 mM KCl 0.15 mM CaCl_2_, 10 mM K_2_HPO_4_/KH_2_PO_4_, 25 mM Hepes, 2 mM EGTA, 5 mM MgCl_2_, 3 mM ATP, 3 mM glutathione). Electroporation was performed in a 4 mm cuvette with 20 µg of linearized pUPRT-RON5-rop5A-Myc, pUPRT-RON5-rop5B-Myc or pUPRT-RON5-rop5C-Myc in a final volume of 800 µl (2 pulses of 1,7 kV for 0,18 ms at 5 sec intervals) (23). After growth for 24 h in HS27 monolayers, transgenic parasites were selected with 5 µM FUDR (5-Fluoro-2′-deoxyuridine, Sigma Aldrich) for at least 4 cycles. Serial dilutions were performed in the presence of 5 µM FUDR.

### Quantification of *rop5* transcripts by qPCR

Total RNA was purified from respective tachyzoites using the Direct-zol™ RNA Miniprep Kit (Zymo Research) according to the manufacturer’s protocol. Complementary DNA (cDNA) was generated for each replicate using the LunaScript RT Supermix (New England Biolabs) based on the manufacturer’s instructions. The cDNA served as template for the amplification of *rop5* genes, using SYBR green I containing Luna^®^ Universal qPCR Master Mix (New England Biolabs). The qPCR was performed using the QuantStudio 5 Real-Time PCR System (Applied Biosystems by Thermo Fisher Scientific). The increase in mRNA expression was determined by the 2-ΔΔCt method relative to the expression of *T. gondii* actin.

### LDH cytotoxicity assay

10 x 10^5^ BL/6 tail fibroblasts were seeded in triplicates in a 96-well plate, stimulated or not for 24 h with 100 U/ml IFN-γ and subsequently infected or not with tachyzoites of indicated *T. gondii* strains at MOI 2. 6 h p.i., Lactamase dehydrogenase (LDH) released from damaged or dying cells into the cell culture medium was quantitatively determined based on oxidation of lactate to generate reduced nicotinamide adenine dinucleotide (NADH). For calorimetric quantification, 50 µl of supernatant (SN) was mixed with 50 µl of reaction mixture containing a yellow tetrazolium salt (INT). After incubation for 30 min at RT, 50 µl STOP solution was added. Conversion by NADH into a red, water-soluble formazan-class dye was measured (LDH Cytotoxicity Assay Kit, Thermo Fisher Scientific) in a microplate reader at OD_492_ nm and corrected by subtracting the absorbance at 680 nm. The intensity of the generated color correlates directly with the number of lysed cells. For a positive control, non-infected cells were lysed in 1x lysis buffer. The percentage of cell death was calculated using the formula: $\%\mathrm{cell death}=(\frac{\text{treated or infected LDH activity - control LDH activity}}{\text{total LDH activity - control LDH activity}})\text{x}100$

### Immunological reagents

Immunoreagents used in this study were: 3E2 mouse anti-ROP5A/B (14, 24), 2.4.21 rat anti-*T. gondii* GRA7 (25), 10E7 mouse anti-Irga6 (26), B34 mouse anti-Irgb6 (27), 940/6 rabbit anti-Irgb10 (25), 2078 rabbit anti-Irgd (28), mouse anti-GBP5 (K. Pfeffer, unpublished), mouse anti-Myc tag (Cell Signaling Technology), mouse anti-HSP90 (Santa Cruz) and mouse anti-actin (Santa Cruz). Alexa 488-labelled goat anti-rat, Alexa 555-labelled donkey anti-mouse, and Alexa 555-labelled donkey anti-rabbit fluorescent antisera (all Life Technologies), goat anti-rabbit-HRP, goat anti-rat-HRP, and rabbit anti-mouse-HRP (all Jackson Immuno Research Laboratories) polyclonal antibodies were used as secondary reagents.

### Immunocytochemistry

Fixation and staining of infected MEFs grown on coverslips was performed as described earlier (29). Microscopy and image analysis was performed blind on coded slides essentially according to (30). Intracellular parasites were identified from the pattern of *T. gondii* GRA7 staining.

### Yeast Two-Hybrid assay

Five single colonies of *Saccharomyces cerevisiae* strain PJ69-4α grown on YPDA (Yeast Peptone Dextrose Agar) plates were resuspended in 100 μl transformation buffer (50% PEG 3350, 0.2 M LiAc, 0.5 mg/ml single-stranded DNA, 0.1 M DTT) and incubated with 1 μg of plasmid DNA (pGAD-C3, pGBD-C3, pGADT7, or pGBDT7 containing the indicated genes) for 30 minutes at 42°C. Cotransformants were selected by plating on double dropout (SD) medium lacking leucine and tryptophan (SD/-Leu/-Trp). Colonies grown on double dropout medium were replica plated on SD/-Leu/-Trp plates. Individual colonies were resuspended in liquid triple dropout medium (SD/-Leu/-Trp/-His), and optical density at 600 nm (OD_600_) determined. Same amount of material was plated on SD/-Leu/-Trp/-His plates supplemented with 0.5 mM 3-aminotriazole (3-AT) and incubated at 30°C for 5 to 14 days.

### Protein-fragment complementation assay

The Protein-fragment Complementation Assay (PCA) (31) was performed as described in (14). Briefly, 7.5 x 10^5^ HEK293T cells were seeded in 6 well-plates and co-transfected 24 h later with 2 µg DNA using Lipofectamine 3000, according to the manufacturer´s instructions. 24 h post transfection, cells were trypsinized, washed 1x with PBS and resuspended in 100 µl passive lysis buffer (1x) containing protease inhibitor cocktail. After 60 min incubation on ice, 50 µl of supernatants (30 min at 15,000 g and 4°C) were mixed with 15 µl nitrocefin and 135 µl PBS in a 96 well-plate. The hydrolysis of nitrocefin was measured by the change of absorbance at 495 nm at intervals of 8–9 seconds for 50 cycles. PCA assays were performed at least three to four times, and differences in average hydrolysis rates were compared to assess interaction strength.

### Lentiviral transduction

For generation of cells stably expressing mCherry-tagged GBP6, plasmids expressing gag-pol and env were co-transfected into HEK293T cells with a plasmid encoding mCherry-tagged GBP6. 24 h post transfection, the medium was exchanged and cells grown for additional 24 h. The supernatant was filtered and transferred onto MEFs that have been seeded 1 day before. After 24 h, transduced cells were selected with 0.5 µg/ml puromycin.

### Plasmid constructs

For Yeast Two-Hybrid analysis, plasmids pGADT7 and PGBKT7 carrying individual *IRG* genes were created earlier (23). *GBP* genes were amplified from modified pWPXL plasmids (32) and cloned into pGADT7 and pGBKT7. For PCA analysis, a DNA fragment encoding ROP5_Chimera_ was amplified from the plasmid pUC-ROP5B-RH-Chimera, synthesized by BioCat, and cloned into BlaN and BlaC. BlaN-Irgb2-b1, BlaC-VAND ROP5B3 and BlaC-RH ROP5B constructs were generated earlier (14). For lentiviral transduction, *GBP6* was amplified from the respective pWPXL plasmid and cloned into pLVX-EF1α-IRES-Puro (Addgene). Table 1 provides an overview of all plasmids and primers generated and/or used in this study.

### Statistical analysis

All statistical analyses were performed using GraphPad Prism 10 software. P-values were determined by an appropriate statistical test. One-way ANOVA followed by Tukey´s multiple comparison was used to test differences between more than two groups. IRG protein frequencies between two groups at *T. gondii*-derived intracellular vacuoles was compared using two-tail t-test. Survival curves were compared by using Log-rank (Mantel-Cox) test. All error bars indicate the mean and standard error of the mean (SEM) of at least three independent experiments.

## Results

### Complementation of *T. gondii* RHΔ*rop5* with single ROP5 isoforms

**Figure 1:**
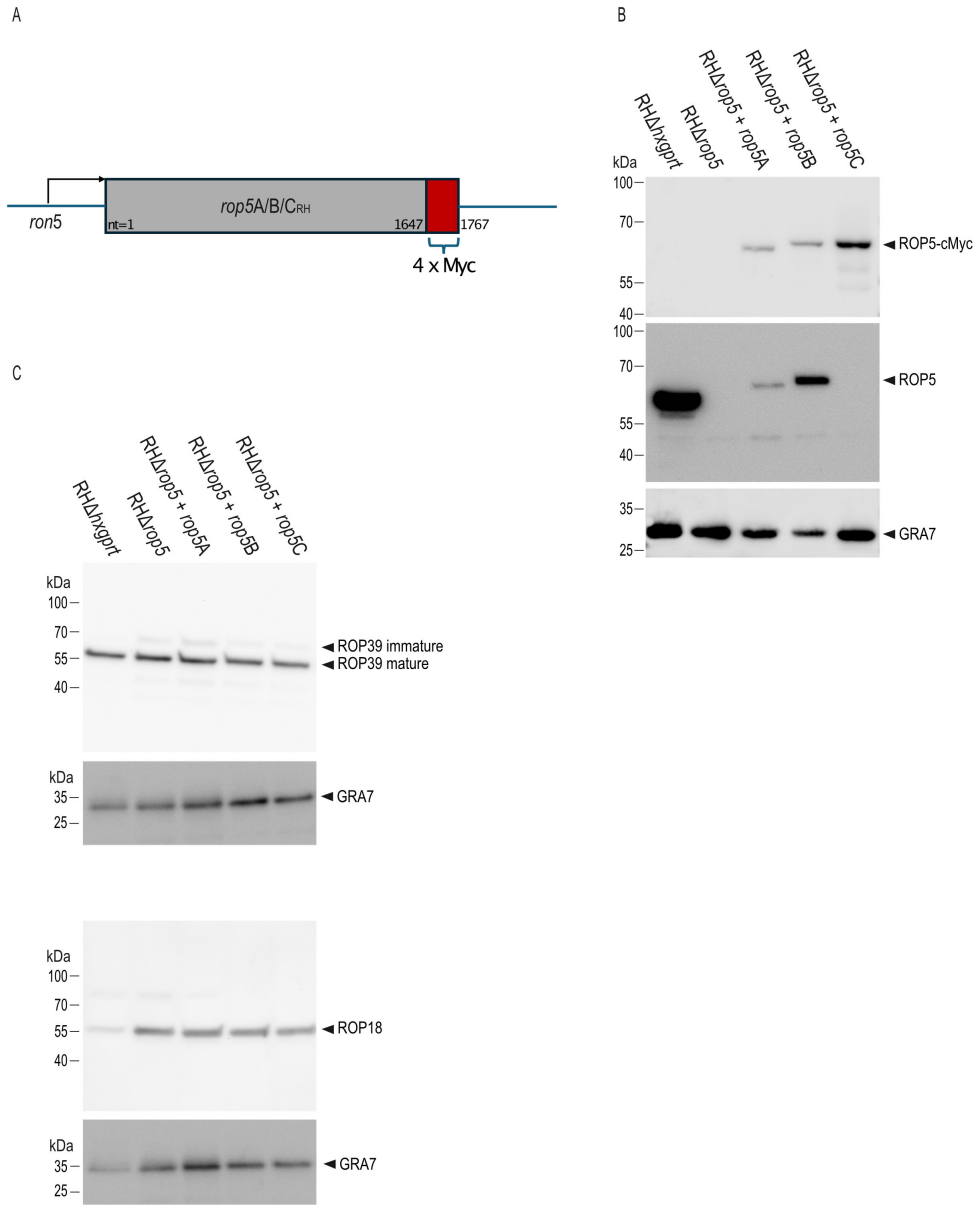
Generation of *T. gondii* RHΔ*rop5* transgenic strains. **(A)**, Schematic representation of *T. gondii* transgenic strains RHΔ*rop5*+*rop5*A, RHΔ*rop5*+*rop5*B and RHΔ*rop5*+*rop5*C, all of which carry a 4x Myc-tag at the C-terminal end (in red). **(B)**, Generation of *T. gondii* trasnsgenic strains is demonstrated on tachyzoite lysates by Western blot using a Myc-tag antibody (upper panel) and an anti-ROP5A/B-specific antibody (middle panel). GRA7 (lower panel) served as loading control. Please note that the ROP5 antibody 3E2 specifically detects ROP5A and ROP5B isoforms, but not ROP5C. This, along with the apparent size shift observed when comparing ROP5A and ROP5B to ROP5C, has been previously reported (14). **(C)**, Expression levels of ROP39 (upper panel) and ROP18 (lower panel) remains unchanged in transgenic strains as demonstrated by Western blot using ROP39- and ROP18-specific antibodies. In both cases, GRA7 served as loading control.

While different combinations of ROP5 isoforms failed to restore full virulence of *T. gondii* in a *rop5* ko background (12, 15), the individual role of ROP5B *in vivo* has not been addressed yet. Here, we generated *T. gondii* strains expressing either ROP5A, ROP5B or ROP5C in a RH*rop5* ko genetic background (**Figure 1A**; **Supplementary Figure 1**). Complementation of the *rop5* ko background with Myc-tagged proteins (RHΔ*rop5*+*rop5*A, RHΔ*rop5*+*rop5*B, RHΔ*rop5*+*rop5*C) was confirmed in tachyzoite lysates by Western blot with an anti-Myc tag antibody (Abcam) (**Figure 1B**, upper panel) and a ROP5-specific antibody (**Figure 1B**, middle panel). GRA7 served as loading control (**Figure 1B**, lower panel). Please note that the ROP5-specific antibody 3E2 detects ROP5A and ROP5B, but not ROP5C, as previously demonstrated (14). Western blot analysis confirms that the expression levels of ROP18 and ROP39 remain unchanged in the complemented strains compared to RHΔ*hxgprt* (**Figure 1C**).

### ROP5B rescues *T. gondii* from IRG-mediated control

**Figure 2:**
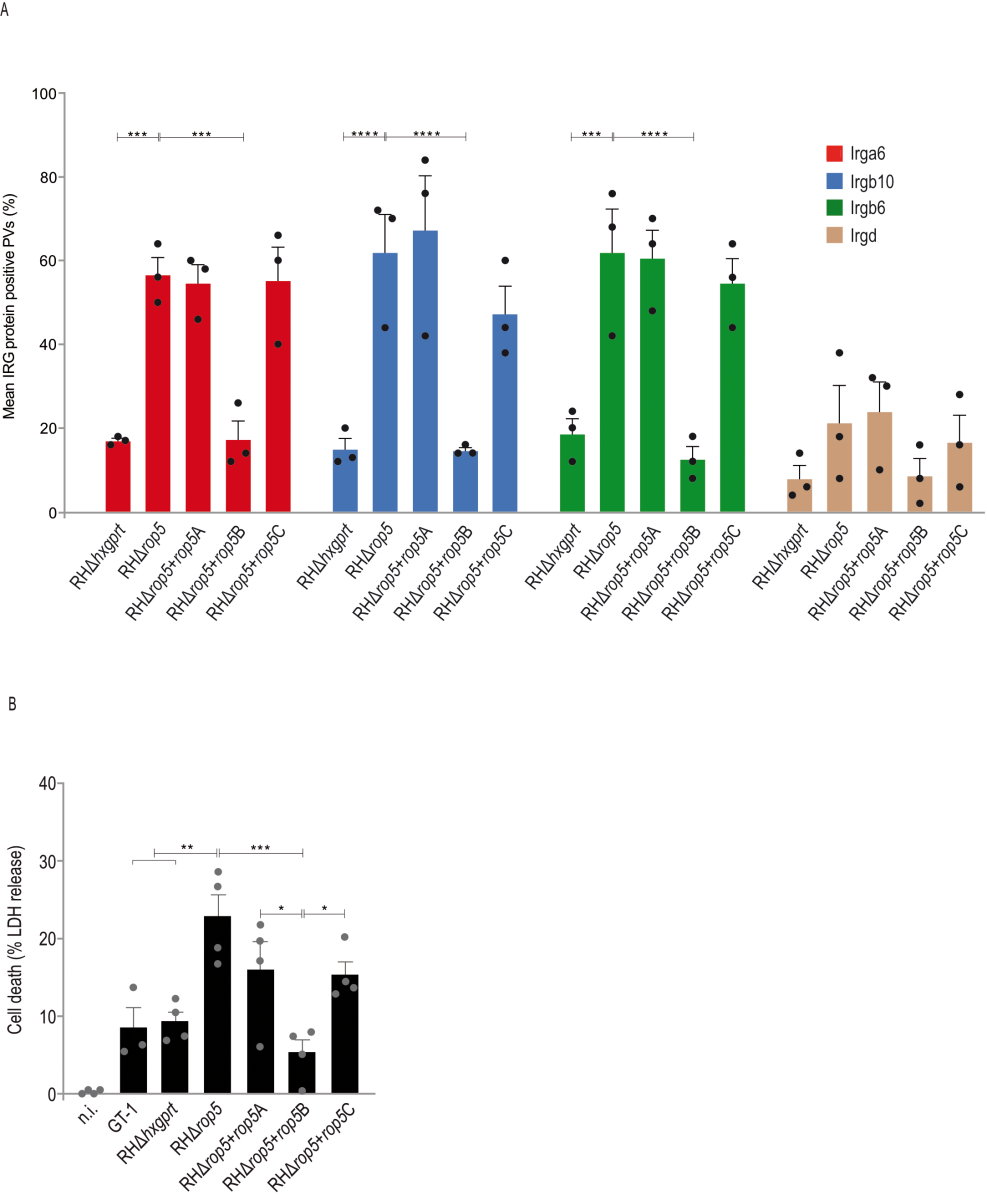
ROP5B but not ROP5A and ROP5C inhibit IRG protein accumulation at the PVM. **(A)**, Mouse embryonic fibroblasts were stimulated with IFN-γ for 24 h and infected (MOI 5) for 2 h before cells were prepared for immunofluorescence. Error bars indicate the mean and standard error of the mean (SEM) of three independent experiments (about 100 vacuoles were evaluated per experiment). Frequencies of vacuoles positive for Irga6, Irgb6 and Irgb10 were significantly decreased in RHΔ*rop5*+*rop5*B compared to RHΔ*rop5* infections and are consistent with the RHΔ*hxgprt* loading phenotype. **(B)**, Cell death, a representative for vacuolar disruption, is significantly decreased in RHΔ*rop5*+*rop5*B compared to RHΔ*rop5* infections. Error bars indicate the mean and standard error of the mean (SEM) of three **(A)** or four **(B)** individual biological replicates, each performed in triplicates. One-way analysis of variance (ANOVA) followed by Tukey´s multiple comparison was used to test differences between groups; ****p< 0.0001; ***p< 0.001; **p< 0.01; *p< 0.05.

*T. gondii* type I strain effectors assemble into complexes that inactivate Immunity-Related GTPases (IRG proteins) at the parasitophorous vacuolar membrane (PVM), with the pseudokinase ROP5 being a central element for complex assembly (9). To assess the role of single ROP5 isoforms in this context, infected C57BL/6 (BL/6) mouse embryonic fibroblasts (MEFs) were analyzed by immunofluorescence. Expression of ROP5B in a *rop5* ko background (RHΔ*rop5*+*rop5*B) reduced vacuolar frequencies of Irga6, Irgb6 and Irgb10 to the same extent like the wt strain whereas frequencies in presence of ROP5A and ROP5C (RHΔ*rop5*+*rop5*A and RHΔ*rop5*+*rop5*C) matched the RHΔ*rop5* loading phenotype (**Figure 2A**).

A hallmark of IRG/GBP-mediated *T. gondii* control is the vesiculation of the PVM, which inevitably and invariably results in the death of the parasite and the death of the host cell (39). To investigate whether the IRG loading phenotype in the presence of ROP5A, ROP5B or ROP5C correlates with IFN-γ-mediated *T. gondii* control, we determined cell death in BL/6 tail fibroblasts by quantification of lactate dehydrogenase (LDH) in the supernatant 6 h post infection. LDH release was significantly increased for RHΔ*rop5*, RHΔ*rop5*+*rop5*A and RHΔ*rop5*+*rop5*C compared to RHΔ*hxgprt*, GT-1 and RHΔ*rop5*+*rop5*B (**Figure 2B**) indicating that only ROP5B but not ROP5A or ROP5C can rescue *T. gondii* from IFN-γ-mediated resistance. These results demonstrate that *T. gondii* ROP5B is required and sufficient to restore parasite virulence in cells of laboratory mice.

### ROP5B is largely responsible for *T. gondii* virulence *in vivo*

**Figure 3:**
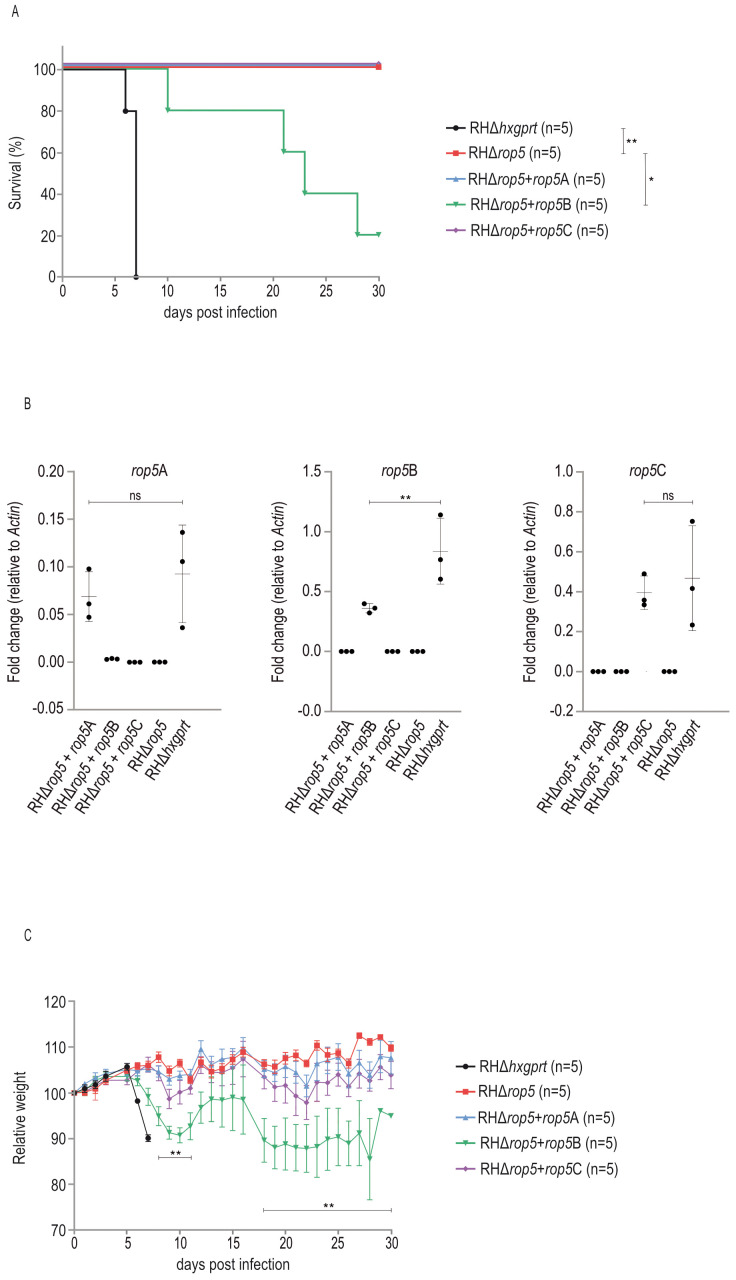
ROP5B restores virulence of *T. gondii* RHΔrop5 *in vivo*. **(A)** Survival of C57BL/6 mice that were intraperitoneally infected with 100 (RHΔ*hxgprt*) or 1000 (RHΔ*rop5*, RHΔ*rop5*+*rop5*A, RHΔ*rop5*+*rop5*B, RHΔ*rop5*+*rop5*C) freshly prepared tachyzoites. Survival curves were compared by using Log-rank (Mantel-Cox) test; **p < 0.01; *p < 0.05. **(B)** Transcripts of *rop5* were quantified by qPCR using isoform-specific primers. Error bars indicate the mean and standard error of the mean (SEM) of three individual biological replicates. One-way analysis of variance (ANOVA) followed by Tukey´s multiple comparison was used to test differences between groups; **p < 0.01; ns not significant. **(C)** Weight loss of mice infected with RHΔ*rop5*+*rop5*A, RHΔ*rop5*+*rop5*B or RHΔ*rop5*+*rop5*C was compared with weight loss of mice infected with RHΔ*rop5* using an unpaired t-test with False Discovery Rate (FDR) correction; **p<0.01.

To evaluate the contribution of single ROP5 isoforms to parasite virulence *in vivo*, we infected BL/6 mice with freshly prepared tachyzoites of *T. gondii* strains RHΔ*hxgprt*, RHΔ*rop5*, RHΔ*rop5*+*rop5*A, RHΔ*rop5*+*rop5*B or RHΔ*rop5*+*rop5*C. Unlike RHΔ*rop5*, RHΔ*rop5*+*rop5*A and RHΔ*rop5*+*rop5*C, RHΔ*rop5*+*rop5*B is lethal in BL/6 animals (**Figure 3A**), indicating a critical role for ROP5B in survival under these conditions. Virulence of RHΔ*rop5*+*rop5*B, however, does not fully phenocopy virulence of the parental strain RHΔ*hxgprt* (**Figure 3A**). In canonical *T. gondii* strains, the *rop5* locus encompasses variable copy numbers of *rop5* paralogs (12, 33). Because the number of individual ROP5 isoforms is not fully restored in the transgenic strains (**Figure 1A**), we determined transcript levels in all strains used in **Figure 3** by qRT-PCR. While *rop5*A and *rop5*C transcript levels remained largely unchanged, *rop5*B transcripts were significantly reduced compared to the wt strain (**Figure 3B**). Lower transcript abundance likely accounts for the defect of ROP5B to fully restore RHΔ*rop5* strain virulence (**Figure 3A**).

Virulence of *T. gondii* is reflected by increased weight loss in case of RHΔ*hxgprt* and RHΔ*rop5*+*rop5*B compared to RHΔ*rop5*, RHΔ*rop5*+*rop5*A and RHΔ*rop5*+*rop5*C (**Figure 3C**).

### The C-terminal polymorphic VAND ROP5B3 surface escapes Irgb2-b1 binding

**Figure 4:**
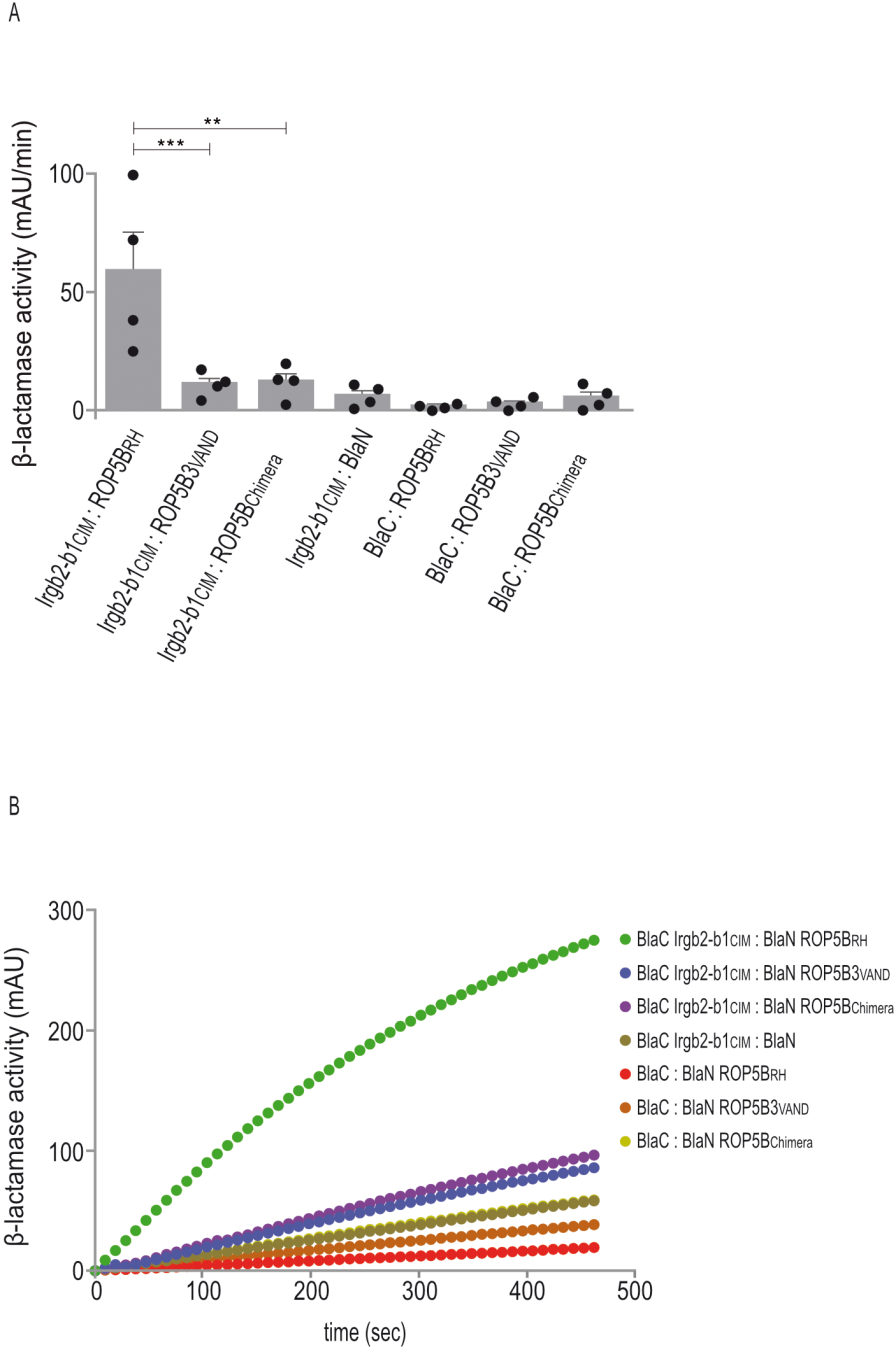
The C-terminal polymorphic ROP5B3_VAND_ surface escapes Irgb2-b1 binding. **(A, B)**, Protein-fragment complementation assay. Proteins were fused to N-terminal (BlaN) or C-terminal (BlaC) fragments of the reporter protein TEM-1 β-lactamase. The increase in absorbance measured at 495 nm indicates restoration of β-lactamase activity after protein:protein-interaction. **(A)**, Binding of Irgb2-b1_CIM_ to ROP5B_RH_ is inhibited in case of ROP5B_Chimera_. Error bars indicate the mean and standard deviation of three independent experiments. One-way analysis of variance (ANOVA) followed by Tukey´s multiple comparison was used to test differences between groups; ***p< 0.001; **p< 0.01. **(B)** The kinetic of the β-lactamase reaction is shown for one representative experiment.

Our own previous data demonstrate that a key molecular mechanism underlying atypical *T. gondii* strain virulence is the escape of ROP5 allelic variants from binding by Irgb2-b1 in CIM cells (14). To confirm these data, we performed Protein-fragment complementation assays (PCA). In that way, binding of CIM Irgb2-b1 to RH ROP5B was completely abrogated in case of VAND ROP5B3 (**Figure 4**) confirming our previous findings from Yeast Two-Hybrid analysis (14).

In our previous study, we generated ROP5B3_VAND_ carrying the polymorphic surface of ROP5B_RH_, thereby restoring binding to Irgb2-b1. We now created a ROP5B_RH_ variant carrying the polymorphic surface of ROP5B3_VAND_ (ROP5_Chimera_) and demonstrate that interaction with Irgb2-b1 is completely inhibited. Binding of Irgb2-b1_CIM_ to ROP5B_RH_ serves as positive control (**Figure 4**). These data indicate, based on *T. gondii* ROP5B3_VAND_, that the highly polymorphic ROP5 surface of atypical strains enables evasion of Irgb2-b1_CIM_ binding and, thus, resistance in CIM mice. They provide further evidence that parasite virulence and mouse resistance may be driven by co-evolutionary dynamics.

### Identification of IRG:GBP pairs

**Figure 5:**
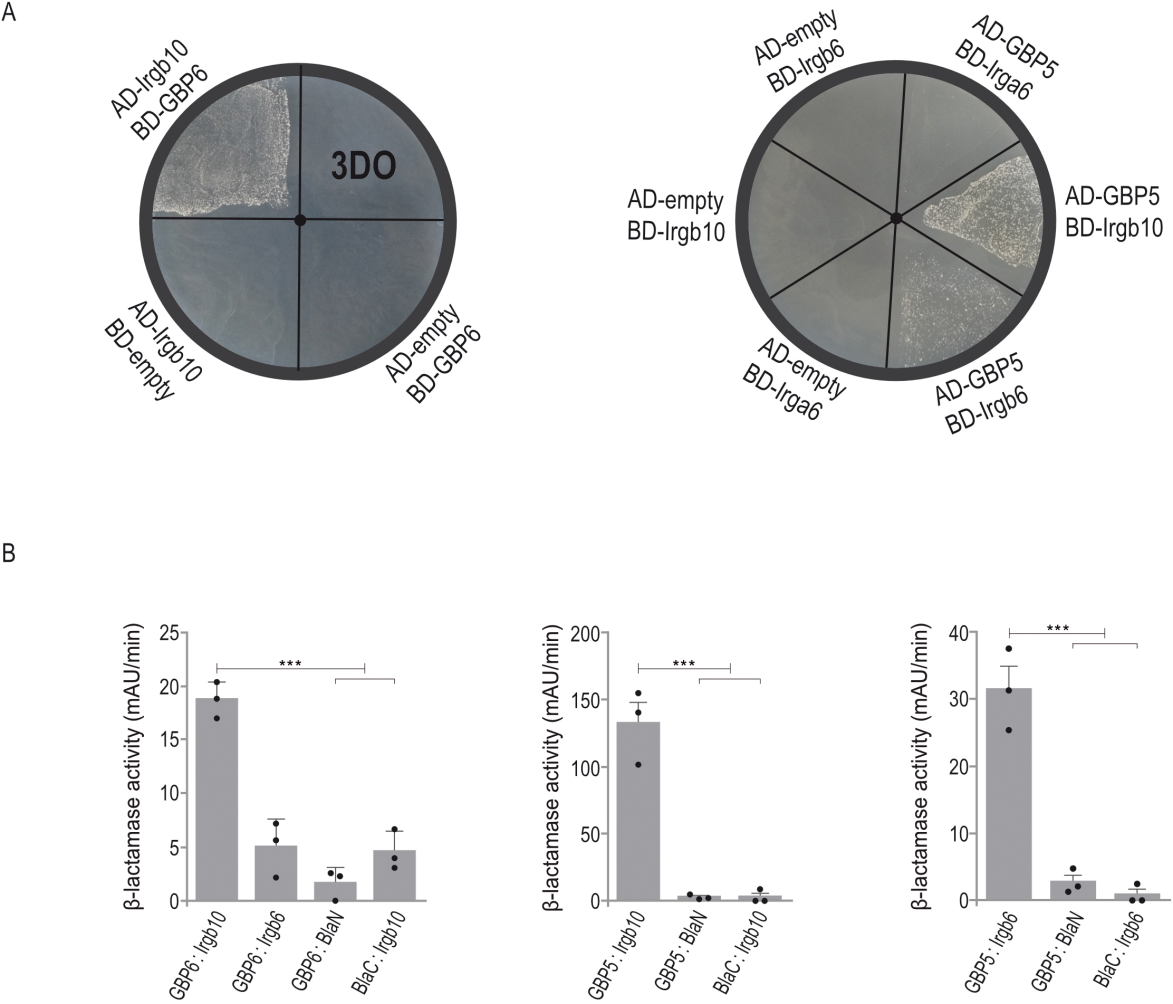
IRG and GBP proteins form heterologous interactions. **(A)**, Yeast Two-Hybrid. Proteins were expressed either as fusion to the Gal4 transcriptional activation (AD) or DNA-binding (BD) domain. Colony growth under 3DO conditions demonstrates GBP6:Irgb6, GBP5:Irgb6 and GBP5:Irgb10 interaction. **(B)**, Protein-fragment complementation assay. Proteins were fused to N-terminal (BlaN) or C-terminal (BlaC) fragments of the reporter protein TEM-1 β-lactamase. The increase in absorbance measured at 495 nm indicates restoration of β-lactamase activity after protein:protein-interaction. Binding of GBP6 to Irgb10 and GBP5 to Irgb6 and Irgb10 confirms YTH results **(A)**. Error bars indicate the mean and standard deviation of three independent experiments. One-way analysis of variance (ANOVA) followed by Tukey´s multiple comparison was used to test differences between groups; ***p< 0.001.

In type I infections, PVM accumulation of IRG and GBP proteins is inhibited by the same parasite effectors. As no effector directly targeting a GBP protein has been identified yet, GBP levels may be indirectly modulated via IRG-specific effectors (9). We propose that an IRG:GBP protein interaction is likely a prerequisite for such an indirect inhibition of vacuolar GBP loading. To gain insight into a possible interdependence between both families of GTPases, we screened various IRG:GBP combinations by Yeast Two-Hybrid (YTH) analysis to identify formation of heterologous binary interactions. IRG and GBP proteins were expressed as N-terminal fusions with the Gal4 DNA-binding (BD) or Gal4 activation domain (AD) in a yeast reporter strain. Colony growth on selective medium is indicative for a direct interaction. In that way, we could identify a direct association of Irgb10 with GBP6 and GBP5 with Irgb10 and Irgb6 (**Figure 5A**). All interactions were confirmed upon over-expression of both proteins linked to different fragments of β-lactamase in HEK cells. The increase in absorbance measured at 405 nm indicates restoration of β-lactamase activity upon protein:protein interaction (**Figure 5B**; **Supplementary Figure 2**). These data suggest the IRG and GBP resistance systems in mice are not independent entities, but might rather be interdependent regarding PVM accumulation and thereby *T. gondii* control.

### IRG/GBP protein accumulation at the *T. gondii* PVM is mutually dependent

**Figure 6:**
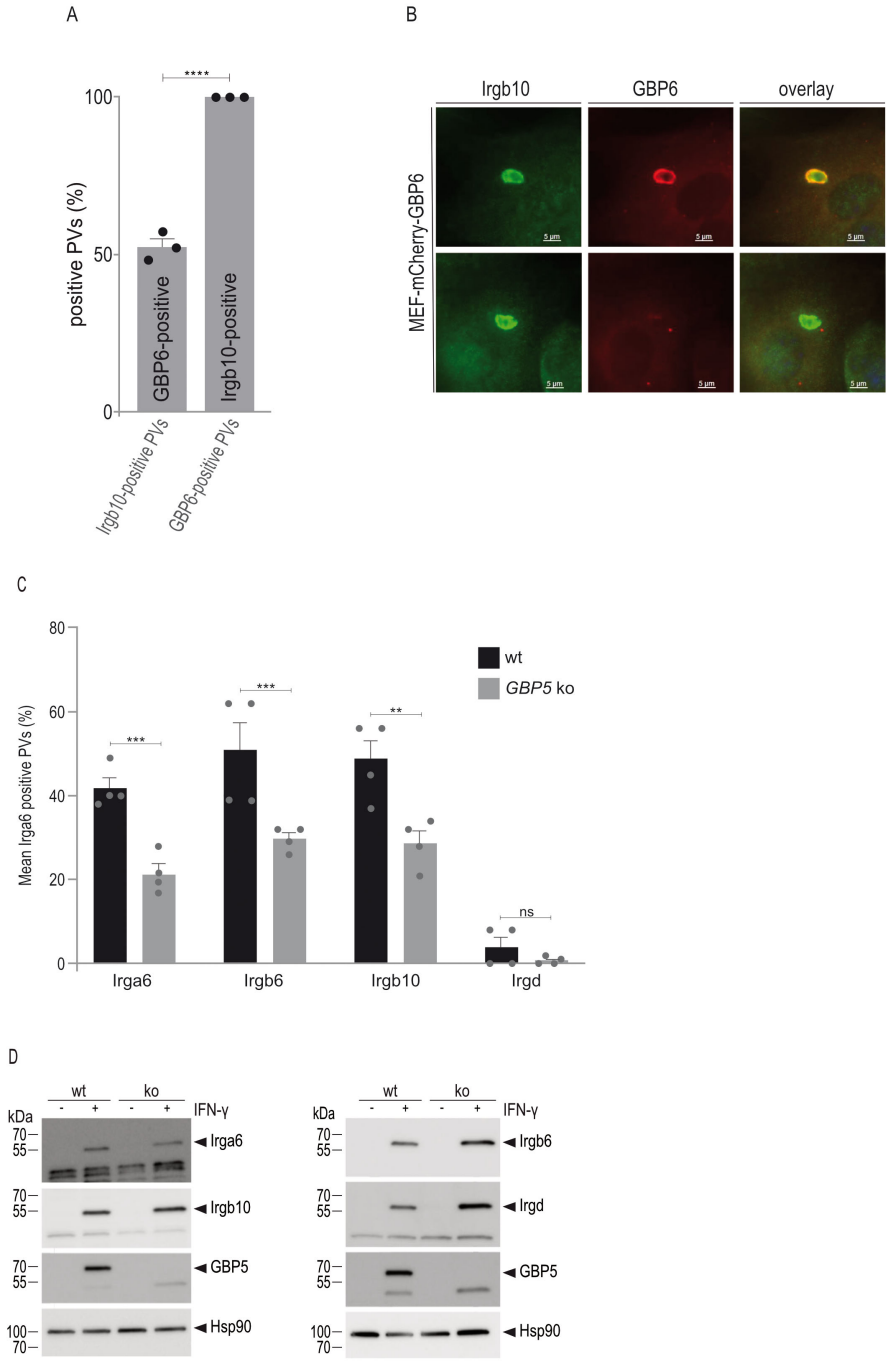
IRG and GBP protein accumulation at the *T. gondii* PVM is mutually dependent. **(A)**, Immortalized mouse embryonic fibroblasts stably expressing mCherry-tagged GBP6 were stimulated with IFN-γ for 24 h and infected for 2 h before cells were prepared for immunofluorescence. Accumulation of GBP6 at the *T. gondii*-derived PVM is dependent on Irgb10. Error bars indicate the mean and standard error of the mean (SEM) of three independent experiments (about 100 vacuoles were evaluated per experiment). A two-tail t-test was used to compre differnces between the two groups; ****p< 0.0001. **(B)**, Representative fluorescent images of *T. gondii* ME49-derived vacuoles, as analysed in **(A)**. **(C)** Mouse embryonic fibroblasts were stimulated with IFN-γ for 24 h and infected for 2 h before cells were prepared for immunofluorescence. PVM accumulation of Irga6, Irgb6 and Irgb10 is significantly decreased in *GBP5* ko compared to wt cells. Error bars indicate the mean and standard error of the mean (SEM) of four independent experiments (about 100 vacuoles were evaluated per experiment). One-way analysis of variance (ANOVA) followed by Tukey´s multiple comparison was used to test differences between groups; ****p< 0.0001; ***p< 0.001; **p< 0.01. **(D)**, Western blot analysis using IRG-specific antibodies demonstrates similar expression levels in wt and *GBP5* ko cells.

After identification of various IRG:GBP pairs (**Figure 5**), we aimed to investigate whether these interactions inform about PVM accumulation of the respective GTPases. Since all our attempts to generate *GBP6* ko cells were unfortunately unsuccessful, we investigated GBP6 and Irgb10 loading onto the *T. gondii* PVM in wt cells after overexpression of mCherry-tagged GBP6. This approach has been successfully applied previously to determine vacuolar GBP frequencies (34). IFN-γ-induced cells grown on coverslips were infected with *T. gondii* ME49 and cells prepared for immunofluorescence 2 h post infection. A total of 300 GRA7-positive vacuoles were evaluated in three biological independent experiments. When we looked for GBP6-positive vacuoles, all of these vacuoles were loaded with Irgb10, whereas only 56% of all Irgb10-positive vacuoles were found to be positive for GBP6 (**Figures 6A, B**). These results indicate that GBP6 accumulation at the PVM is likely dependent on Irgb10.

The significance of a GBP5 interaction with Irgb6 and Irgb10 for vacuolar accumulation of these GTPases was investigated in *GBP5* ko cells. In the absence of GBP5, frequencies of Irga6, Irgb6 and Irgb10 are significantly reduced (**Figure 6C**). Expression levels of these IRG proteins are similar to those in wt cells (**Figure 6D**) and therefore do not account for the reduced vacuolar loading frequencies. While inhibition of Irgb6 and Irgb10 loading can be attributed to the absence of GBP5, the inhibition of vacuolar Irga6 accumulation - for which we did not find an interaction with GBP5 (**Figure 5**) - is likely due to an IRG loading hierarchy. In this regard, the dependence of Irga6 accumulation on Irgb6 has been demonstrated (30). Altogether, these results indicate that IRG and GBP loading onto the PVM is mutually dependent and regulated through pairwise heterologous interactions of different members of both GTPase families.

## Discussion

*T. gondii* virulence in mice depends on effector proteins that are secreted from apical organelles into the host cell cytosol. In a type I x II (13) and type II x III (12, 35) genetic cross, the pseudokinase ROP5 emerged as one major QTL (quantitative trait locus) in laboratory strains of mice. The importance of ROP5 extends beyond the clonal lineages dominating in North America and Europe and is also applicable to different isolates from South America (SA) and Asia (17), despite considerable genetic differences between these strains (6, 36). These findings indicate the conservation of important functions of ROP5 for virulence across genetically diverse *T. gondii* strains.

We here show that ROP5B mainly contributes to *T. gondii* type I strain virulence *in vivo*. The difference upon complementation relative to the wt strain is probably due to lower *rop5*B transcript abundance (**Figure 3**) and, thus, lower ROP5B expression levels. The transcript levels of *rop5*A and *rop5*C remain unchanged upon complementation compared to the wt strain (**Figure 3B**), suggesting that the defect in virulence of ROP5A and ROP5C, compared to ROP5B (**Figure 3A**), is not due to the expression levels of these two ROP5 isoforms but rather arises from differences in their protein functions. However, we cannot completely exclude the possibility that ROP5A and/or ROP5C contribute to parasite virulence in a synergistic function with ROP5B.

The importance of ROP5B for parasite virulence implicates that it forms - in contrast to ROP5A and ROP5C - distinct interactions that are essential for *T. gondii* to manipulate the host immune response. In this regard, ROP5 has been shown to act as scaffolding element, facilitating the assembly of distinct multiprotein complexes at the PVM, each targeting a specific Immunity-Related GTPase (IRG protein). Until now, three rhoptry proteins have been demonstrated to interact with ROP5 (9). While ROP5B is the isoform in case of ROP18 and ROP39 complexes, the ROP5 isoform involved in ROP17 complex formation remains to be identified. Besides its role as scaffolding element for *T. gondii* effectors, ROP5 also directly acts on the IRG system. ROP5B and ROP5C use a C-terminal polymorphic surface to target Irga6 adjacent to the nucleotide-binding domain (37, 38), inducing allosteric changes in the Irga6 structure that result in exposure of two highly conserved threonine residues in the switch I region that are targeted by ROP18 (37). In addition, ROP5B or ROP5C binding covers an interface necessary for Irga6 oligomerization (37, 38) which is a prerequisite for membrane rupture (26, 39). The failure of ROP5C to complement *T. gondii rop5* ko strain virulence (**Figure 3A**) might be due to the lack of allosteric activation of the IRG-related active kinases. While a ROP5B activity towards ROP18 has been shown in this regard (29), ROP5C and ROP5A have not yet been tested.

The predominant role of ROP5B in parasite virulence is further supported by the molecular mechanism that provides resistance to *T. gondii* in wild-derived mice. In cells derived from CIM (*Mus musculus castaneus*) mice, a polymorphic Irgb2-b1 variant (16) directly binds and sequesters ROP5B, thereby conferring resistance even against type I strains (14). In SA, *T. gondii* strains have been isolated that are genetically highly diverse and associated with high mortality rates in mice. We could demonstrate that SA *T. gondii* strains are virulent in CIM mice because polymorphic ROP5 variants escape binding by Irgb2-b1, exemplified by *T. gondii* VAND ROP5A/B1/B2/B3 (14). On that basis, we have now replaced the amino acids constituting the interface for Irgb2-b1 binding in ROP5B_RH_ (14) with the respective region from ROP5B3_VAND_ (ROP5_Chimera_) and investigated binding to Irgb2-b1_CIM_. In the PCA, binding of ROP5_Chimera_ to Irgb2-b1 is completely abolished (**Figure 4**). Since we have shown that VAND ROP5A/B1/B2 also do not interact with Irgb2-b1 (14), the C-terminal polymorphic surface likely serves as the element for evading CIM-inherited resistance across all VAND isoforms.

Recently, we have identified ROP5 and ROP18 as inhibitors of IL-1β secretion upon *T. gondii* infection. ROP5A and ROP5B interact with mouse GBP5 and thereby inhibit NLRP3 complex assembly (20). This is the first evidence of a direct interaction between a *T. gondii* effector and a GBP protein. Regarding the loading of the PVM with GBP5, however, present results are inconsistent. While three studies initially detected almost no GBP5 accumulation (34, 40, 41), two subsequent investigations reported high frequencies of type II-derived GBP5-positive vacuoles (42, 43). Here, we identified direct binding of GBP6 to Irgb10 and GBP5 to Irgb6 and Irgb10 and demonstrated that accumulation of the respective GTPases at the PVM is mutually dependent. Although PVM localization of Irgb6 and Irgb10 depend on GBP5, we were unable to detect any GBP5-positive vacuoles (data not shown). The accumulation of the respective IRG proteins may be regulated by GBP5, even though GBP5 itself may not be localized to the PVM. Considering all available data, it appears more likely, however, that GBP5 is crucial for parasite virulence, both at the PVM and beyond. In this scenario, ROP5B may inhibit GBP5 accumulation at the vacuole, further underscoring the predominant role of this ROP5 isoform in *T. gondii* virulence, while ROP5A mediates the inhibition of the GBP5:NLRP3 interaction (20). Both, the localization of GBP5 and the specific inhibitory roles of ROP5A and ROP5B, still need to be further investigated.

The *rop5* gene cluster is represented by a varying number of genes, depending on the *T. gondii* strain (12, 13, 33), that encode three different isoforms. Interestingly, type II *rop5*B harbors a frameshift mutation resulting in a truncated protein (12, 33). Since the *rop5*C coding sequence is very similar to *rop5*B (eight nonsynonymous substitutions), it was assumed that a predicted non-functional ROP5B is unlikely to be the cause of virulence differences between canonical strains. Furthermore, the expansion of *rop5*C gene copy numbers is especially pronounced in the type II locus, suggesting that these additional genes might compensate for a defective ROP5B version. However, our results, which highlight a predominant role for ROP5B, suggest that this frameshift mutation could explain the attenuated virulence of *T. gondii* type II strains.

The expansion and diversity of the *rop5* locus may indicate that other allelic products than ROP5B might have co-evolved with IRG proteins or other host cell resistance molecules in evolutionary important intermediate hosts beyond *Mus musculus*. Rodents serve as the primary prey of domestic cats, making them an important intermediate host in the parasite´s life cycle (44, 45). Nevertheless, not all prey animals pose the same risk of infection for cats, considering variations in seroprevalence and behavioural and ecological differences between rodent species (45, 46). Moreover, the feeding behavior of cats plays a crucial role in this context. The prey composition of domestic cats (*Felis catus*) closely mirrors that of feral cats (*Felis silvestris*). This European subspecies primarily feeds on small rodents, such as wood mice (*Apodemus sylvaticus*) and voles like *Myodes glareolus* (47). Persistently infected local rodent species probably contribute to parasite transmission to domestic cats, leading to environmental oocysts contamination, a prerequisite for spillover into livestock and humans. An important area for future research is to determine the molecular mechanism of *T. gondii* resistance in intermediate hosts other than *Mus musculus* - with a particular focus on Irgb2-b1 - and to investigate which, if any, ROP5 isoforms play a pivotal role in the infection biology of the parasite in these species.

Deciphering the specific roles of individual parasite effectors is essential for our understanding of *T. gondii* virulence and for identifying potential targets for therapeutic strategies. Moreover, studying pseudokinases, like ROP5, enhances our comprehension of the diverse roles and functions of these seemingly dead enzymes in biology, evolution, and disease, further challenging our view on kinase activity and catalysis.

## Data Availability

The raw data supporting the conclusions of this article will be made available by the authors, without undue reservation.

## Supplementary material

Supplementary Figure 1 Generation of complemented *T. gondii* RHΔ*rop5* strains. Schematic representation of specific gRNA-mediated targeting of CRISPR/Cas9 to the endogenous *UPRT* locus and integration of C-terminally Myc (4x)-tagged *rop5*A, *rop5*B or *rop5*C carrying 5`and 3`UTR homology regions.

Supplementary Figure 2 Protein-fragment complementation assay. The kinetic of the β-lactamase reaction for PCA assays in **Figure 5B** is shown for one representative experiment respectively.

Supplementary Table 1 Primers, plasmids, antibodies and reagents used in this study.
