# Supplementary figures and images for: Toxoplasma gondii virulence in mice is determined by the pseudokinase ROP5B and countered by an IRG-GBP protein interplay
Source: Front Immunol. 2025 Jul 9;16:1593785. doi: 10.3389/fimmu.2025.1593785 (PMC12283572; doi:10.3389/fimmu.2025.1593785)

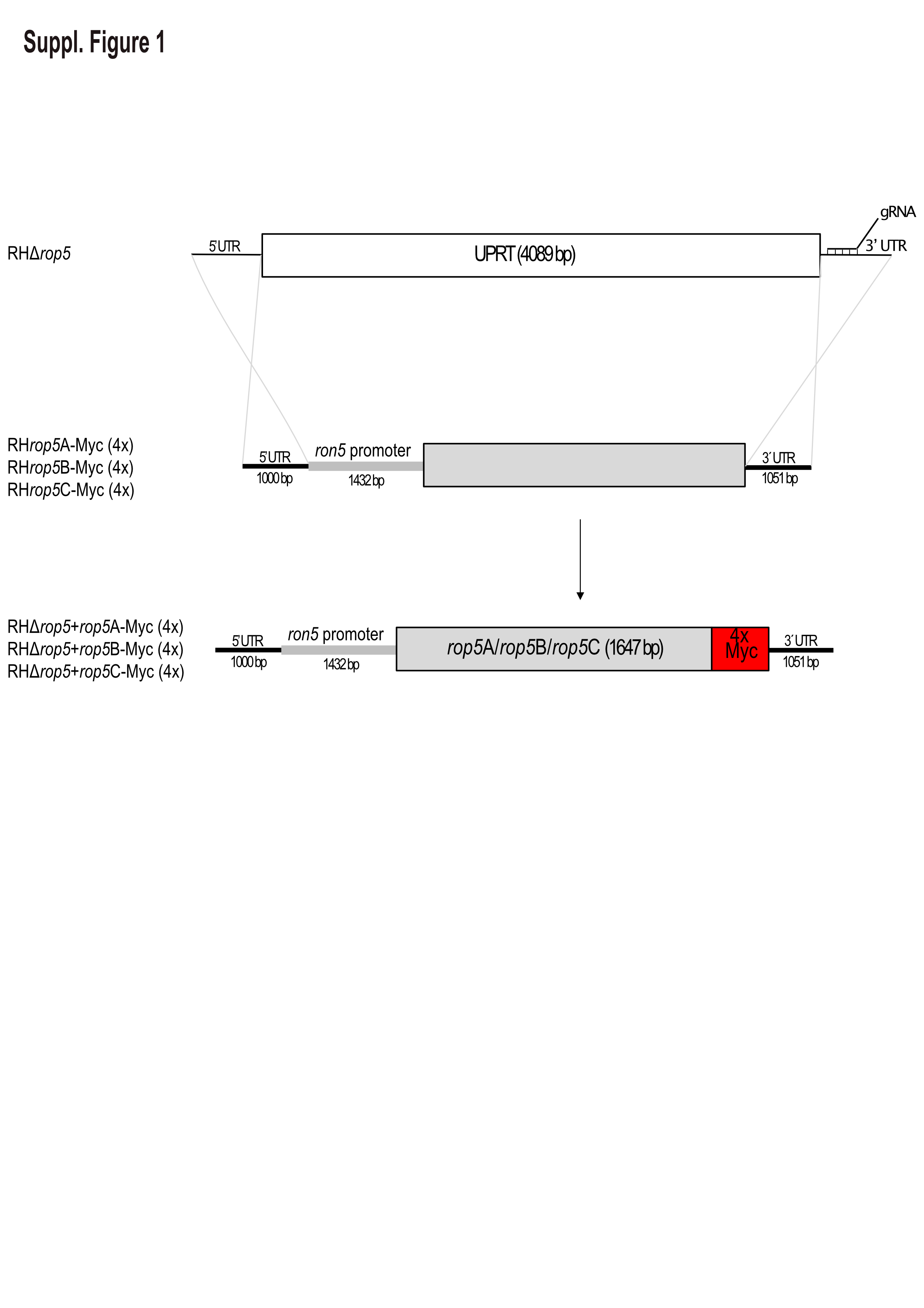

Supplement: Supplementary Figure 1 — Generation of complemented T. gondii RHΔrop5 strains. Schematic representation of specific gRNA-mediated targeting of CRISPR/Cas9 to the endogenous UPRT locus and integration of C-terminally Myc (4x)-tagged rop5A, rop5B or rop5C carrying 5`and 3`UTR homology regions. [file Image1.jpg]

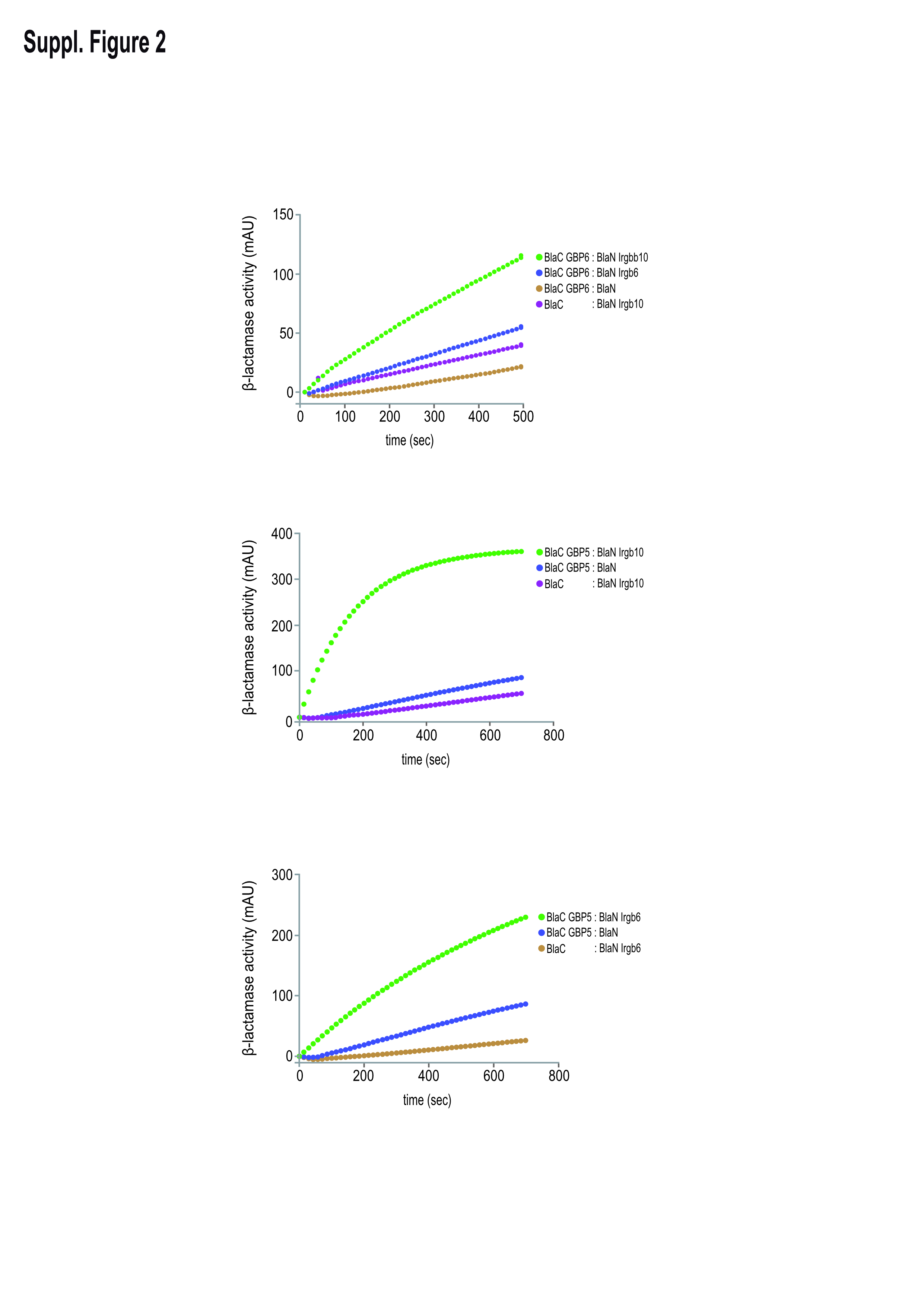

Supplement: Supplementary Figure 2 — Protein-fragment complementation assay. The kinetic of the β-lactamase reaction for PCA assays in Figure 5B is shown for one representative experiment respectively. [file Image2.jpeg]
